# Effects of Synthetic Diets Enriched in Specific Nutrients on *Drosophila* Development, Body Fat, and Lifespan

**DOI:** 10.1371/journal.pone.0146758

**Published:** 2016-01-07

**Authors:** Tânia Reis

**Affiliations:** Department of Medicine, Division of Endocrinology, Metabolism and Diabetes, University of Colorado Medical School, Anschutz Medical Campus, Aurora, Colorado, United States of America; Lancaster University, UNITED KINGDOM

## Abstract

Gene-diet interactions play a crucial but poorly understood role in susceptibility to obesity. Accordingly, the development of genetically tractable model systems to study the influence of diets in obesity-prone genetic backgrounds is a focus of current research. Here I present a modified synthetic *Drosophila* diet optimized for timely larval development, a stage dedicated to energy storage. Specifically increasing the levels of individual macronutrients–carbohydrate, lipid, or protein–resulted in markedly different organismal effects. A high-carbohydrate diet adversely affected the timing of development, size, early lifespan and body fat. Strikingly, quadrupling the amount of dietary lipids had none of these effects. Diets rich in protein appeared to be the most beneficial, as larvae developed faster, with no change in size, into long-lived adults. I believe this synthetic diet will significantly facilitate the study of gene-diet interactions in organismal energy balance.

## Introduction

Obesity rates are high and continue to climb. There are clearly genetic factors at play, but we also know that dietary trends contribute to the development of the disease. Multiple animal models have been developed in order to try to better understand the onset and development of obesity and derived metabolic disorders, the complexity of which present profound challenges to research. Gene-diet interactions (i.e., how genetic background influences an individual’s response to a given diet) are even more complex, illustrated by the difficulty in predicting outcomes of interventional diets [1]. In general, the dietary components considered relevant to weight management are lipids, carbohydrate, and protein. Manipulating both the relative and absolute amounts of these components in the diets of model organisms leads to a variety of effects on body weight, body fat, overall metabolism, and longevity, among other biological parameters [1]. However, simultaneously varying the genetic background to identify the underlying molecular pathways is only feasible in models amenable to unbiased genetic screens.

*Drosophila melanogaster* has a long history as an experimental model for the study of various human diseases. In terms of metabolic disorders, fruit flies features fundamentally similar overall physiology and regulatory organs that functionally parallel those of mammals. Several studies have identified genetic lesions that lead to “obese” flies with excess body fat [2–5]. A number of these genes have conserved functions in mammals [3, 5]. In addition to genetic dissection, several studies have examined interactions between diet and metabolism-related parameters including diabetes, heart disease, and lifespan [6–10]. Typically, to modulate protein content, levels of dietary yeast are varied [10], whereas fat levels are often increased by using coconut oil [9], and extra sucrose is added to increase carbohydrates [7, 8]. Although yeast is the major source of protein in standard fly stock media, yeast is clearly more complex than just protein, and coconut oil is a complex fat. There is thus a need for synthetic *Drosophila* diets to more precisely manipulate individual macronutrients, allowing dissection of gene-diet interactions in the context of organismal metabolism. Toward this goal, several recent studies have described distinct synthetic diets [11–14]. However, these diets induce drastic delays in larval development [12, 13] (Table 1). Energy stored during the larval stages fuels metamorphosis and much of early adult life [15]. Accordingly, suboptimal nutrition during development complicates the analysis of gene-diet and metabolism interactions.

**Table 1:** Concentrations (in g/L) of relevant components of various *Drosophila* media and relevant indicators of dietary effects on development, fecundity/fertility, and longevity.

Diet	Protein	Carbs	Lipid	Vit & Min^a^	Gel^b^	Other^c^	Dev. Timing (days)	Life- span^d^	Ref.
Synthetic	73.3	13.3	0.40	9.8	5	1.7	10.36 (adult eclosion)	63	This study
Holidic (50S200N HUNTaa)	21.4	17.1	0.1–0.3	36.0	20	7.5	9.9 (to first pupa)	~55	[13]
Chemically Defined (400K)	19.6	78.4	0.87	3.2	10	–	13.2 (adult eclosion)	37	[12]
Sang’s medium C	55.0	7.5	0.3	6.2	30	0.02	n.d.^e^	n.d.	[16, 17]
Hunt’s medium	24.5^f^	7.5	0.3	6.2	30	0.02	n.d.	n.d.	[16]
Bloomington stock recipe	19.0	63.9	2.4	1.2	5.3	5.7	n.d.	n.d.	^g^
“Regular Food”	24.3	132.5	16.8	1.9	9	1.9	8.6 (adult eclosion)	35	[12]

^a^Vitamins, minerals and nucleic acids

^b^Gelling agent

^c^Preservatives, etc.

^d^Median lifespan for females.

^e^“n.d.”, not determined.

^f^Hunt’s medium is derived from Sang’s “defined” medium but utilizes individual amino acids instead of casein.

^g^Based on nutritional contents of indiviual ingredients as provided by their manufacturers; see Bloomington Stock Center website (http://flystocks.bio.indiana.edu/Fly_Work/media-recipes/bloomfood.htm).

In this study I develop a synthetic *Drosophila* diet on which larvae develop at rates approaching those observed on rich diets. I determine the effects on development, size, total body fat levels, feeding behavior and lifespan of increasing levels of specific macronutrients (carbohydrates, lipids or protein). On a carbohydrate-rich diet, larvae have elevated triacylglycerides (TAGs), develop more slowly into smaller pupae, and, as adults, display changes in age-associated mortality. By contrast, on a protein-rich diet, larvae do not accumulate extra TAGs, and develop faster with no changes in size when compared to their sibling controls fed a normal diet. These findings emphasize the requirement of balanced diets for optimal development, growth and lifespan.

## Materials and Methods

### Fly stocks and husbandry

Wildtype Oregon R flies were originally obtained from the Bloomington Stock Center (stock #4269) and have been maintained in the lab for several generations. Adult flies were allowed to lay eggs on grape juice plates with yeast paste at 25° for 4–6 hr. 24 hr after hatching, 50 first-instar larvae were transferred to each vial of synthetic medium. All experimental animals were maintained in a 25° incubator.

### Synthetic media food

The synthetic diet used here has been modified from Sang’s “defined” medium for *Drosophila* culture [16] to optimize for developmental timing and eclosion rates. The concentration of sucrose was increased 1.75-fold and that of all ingredients was increased 1.33-fold relative to the original recipe, and the agar content was reduced (Table 1). Normal medium (N), per liter: 5 g agar (VWR, 90000–760), 73.3 g casein (Sigma, C5679), 13.3 g of sucrose (Fisher Scientific, S5), 0.4 g cholesterol (Sigma, C4951), 0.32 g choline chloride (Sigma, C1879), 0.85 g inosine (Sigma, I4125), 0.76 g uridine (Sigma, U3003), 133.3 ml NaHCO_3_ (10 g/L stock), 133.3 ml KH_2_PO_4_ (7.1 g/L stock), 133.3 ml K_2_HPO_4_ (37.3 g/L stock), 133.3ml MgSO_4_ • 7 H_2_O (6.2 g/L stock), 133.3 ml vitamin A stock, 13.3 ml vitamin stock B stock, 333 ml deionized water, and 13.3 ml 10% Tegosept (weight/volume in 95% ethanol; Genesee Scientific, 20–259). Vitamin A stock was 20 mg thiamine (Sigma, T1270), 100 mg riboflavin (Sigma, R4500), 120 mg nicotinic acid (Sigma, N0761), 160 mg D-pantothenic acid hemicalcium salt (Sigma, P5155), 25 mg pyridoxine (Sigma, P6280) and 2 mg biotin (Sigma, B4501), dissolved in 1 L deionized water, aliquoted and frozen at -20°C protected from light. Vitamin B stock was 500 mg folic acid (Thermo Fisher, BP2519) dissolved in 167 ml 20% ethanol, aliquoted and frozen at -20°C protected from light. To make 4 times carbohydrate (4C), 4 times protein (4P), or 4 times lipids (4L) medium, sucrose, casein, or cholesterol and choline chloride were increased to 53.2 g, 293.2 g, or 1.6 g and 1.28 g, respectively. Notably, the casein product used (Sigma, C5679) is a non-homogenous mixture of polypeptide variants, the exact composition of which depends on genetic polymorphisms in the cows from which it was obtained [18]. However, these variants are ~99% identical; the product thus contains 87–94% protein whose amino acid composition is ~99% defined. Media were autoclaved for 30 min using a liquid cycle and Tegosept was added only after autoclaving. To prevent settling of casein, the media were stirred while cooling and prior to pouring.

### Triglyceride and protein measurements

25 Oregon R larvae of each experimental condition were homogenized in a microcentrifuge tube using a pestle and centrifuged for 5 min at 13,200 rpm. 8 μl of supernatant was transferred to a fresh tube and centrifuged again for 5 min at 13,200 rpm. 5 μl of this sample was used in the TAG assay using a Triglyceride Reagent Kit (Sigma, T2449), per manufacturer’s instructions. 1 μl of sample was used in the Protein Assay Kit II (BioRad, 500–0002) as recommended by the manufacturer. Levels of TAGs were normalized to levels of soluble protein. Three independent assays (of 25 larvae each) were performed for each experimental condition. Graphpad Prism v6 software was used to perform statistical tests.

### Nile Red staining

Larvae were inverted to expose fat bodies and fixed with 8% paraformaldehyde (Electron Microscopy Sciences, 15710) for 45 min. Dissected fat bodies were washed three times in PBTriton 0.1% and stained with 0.25 μg/ml Nile Red (Molecular Probes, N1142) in PBS for 30 min. After washing three times in PBTriton 0.1%, fat bodies were mounted on slides in SlowFade mounting media (Molecular Probes, S36936) to decrease photo-bleaching. Images were obtained using a confocal microscope.

### Size measurements

Pupae from larvae reared on the specified diets were measured using a caliper. 54 pupae were measured for each experimental condition. Graphpad Prism v6 software was used to perform Ordinary one-way ANOVA test.

### Eclosion rates

Eclosion values were measured for each vial as the duration (in days) until the first adults eclosed from pupal cases. Values were recorded daily in the morning and evening. At least 230 animals (7 vials with ~50 animals each) were analyzed for each experimental condition.

### Food intake

Larval food intake was measured as described previously [3], except that larvae reared on the experimental diets were exposed for 30 min to the same diet to which colored dye was added. Three independent assays were performed for a total of 60 animals per experimental condition, and Graphpad Prism v6 software was used to perform Ordinary one-way ANOVA test.

### Life span analysis

Larvae seeded on the specific diets were allowed to eclose for 24 hr, and then to mate for 48 hr. Females were transferred into fresh vials of the same diets on which they were grown. Death events were recorded daily. At least 300 flies were analyzed per experimental condition, using Graphpad Prism v6 software to perform statistical analysis (Log-rank (Mantel-Cox) and Gehan-Breslow-Wilcoxon tests). Flies were transferred to vials with fresh media every 2–3 days. The old vials were kept and animals eclosing from eggs that were laid in them were evaluated to assess fertility.

## Results

As a first step towards dissecting the role of dietary composition in organismal energy balance, I developed enriched diets based on a recipe for synthetic fly food [16] (see Materials and Methods). Sang’s “defined” medium for *Drosophila* culture induces significant delays in development–up to 2 days delay in larval wandering compared to lab stock food–and low rates of eclosion (data not shown). I noticed that increasing the concentration of any single component of Sang’s defined diet (i.e., carbohydrate, lipid, or protein) accelerated larval development and increased eclosion rates (unpublished observations). Accordingly, I hypothesized that each of these components is somewhat limiting in Sang’s “defined” diet, and tested modified recipes in which the concentrations of all ingredients were increased to various extents (1.33- to 2.67-fold) relative to the original recipe. These media were of a more solid consistency due to the decreased concentration of water, so I reduced the concentration of gelling agent (agar) (Table 1). I found that, while larvae developed the fastest on a diet with 2.67x more sucrose and 2x more of the other components, on a diet with ~1.75x more sucrose and ~1.33x more of the other components (Table 1) the duration to wandering was comparable to molasses/yeast-based stock food and rates of eclosion were the highest (data not shown, and see below).

Next, I specifically increased the relative abundance of lipid, carbohydrates, or protein, and monitored the effects on various parameters of energy homeostasis in larvae and adults. Wildtype first instar (L1) larvae were grown on four different diets: Normal (N), and derivatives of N in which the concentration of a particular macronutrient–lipids (4L), carbohydrates (4C), or protein (4P)–was quadrupled. Embryos were collected on grape plates, allowed to hatch and L1s were transferred to the respective medium and allowed to develop until wandering or adult stages.

**Fig 1:**
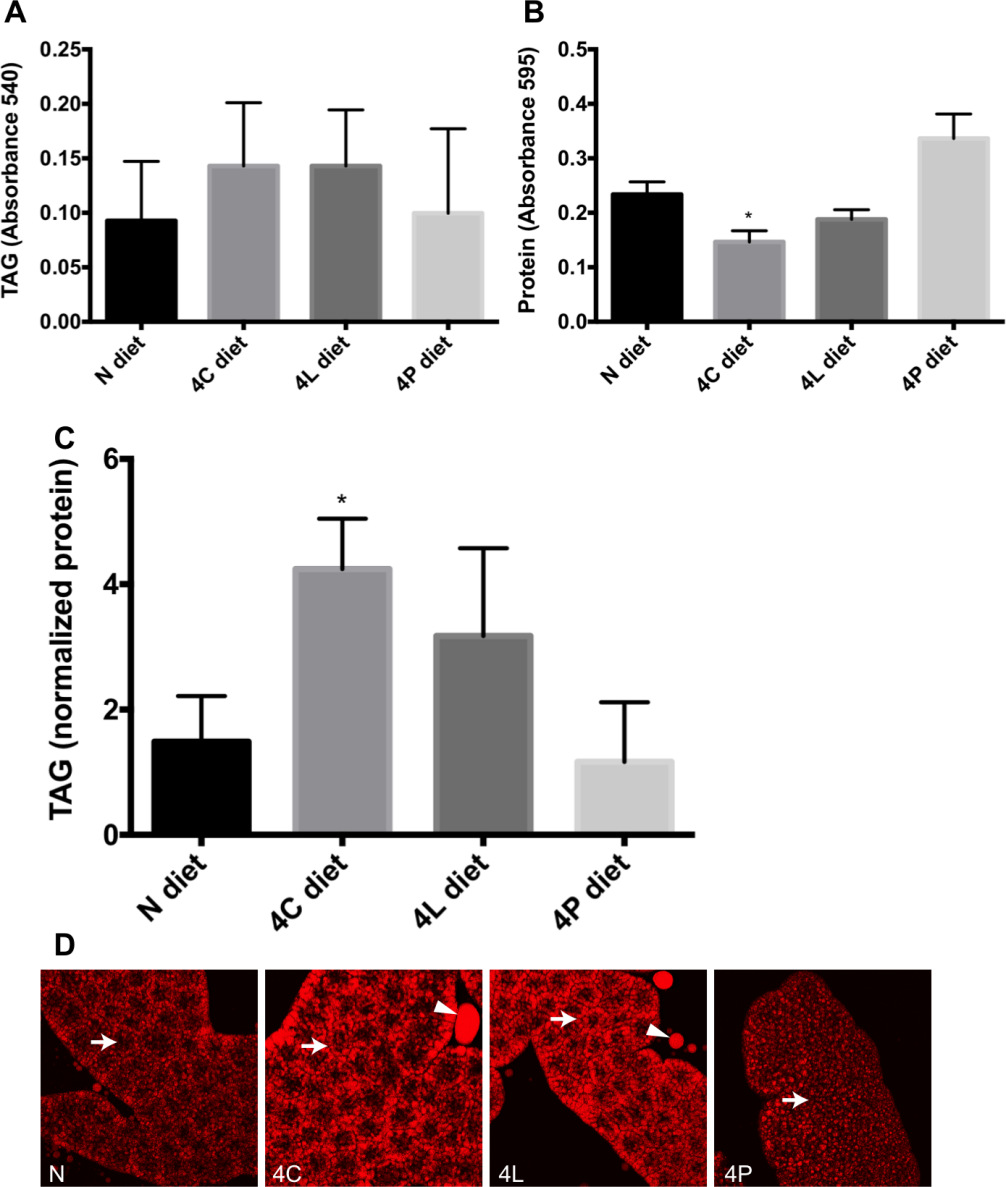
Effect of enriched diets on fat accumulation. Larvae were fed a balanced (N), carbohydrate-rich (4C), lipid-rich (4L) or protein-rich (4P) diet. (A-C) Larval extracts were analyzed for (A) total TAGs, (B) total soluble protein, or (C) total TAGs normalized for soluble protein. (D) Fat bodies of larvae were stained post-fixation for the fat-specific dye Nile Red (red). Fat bodies of larvae grown in 4C and 4L diets have higher levels of fat per cell (arrow), and commonly display “leakage” of fat from the isolated tissue (arrow head). Asterisks indicate significant differences (P < 0.05) for Ordinary one-way ANOVA. Error bars, standard deviation. Since in (C) the variances appeared unequal, both a nonparametric test (Mann Whitney) and a t test with Welch’s correction for unequal variances were also applied to these data. The Mann Whitney test found no significant difference (all P > 0.05) between the diets, whereas the Welch-corrected t test found significance only for the N-to-4C comparison (P = 0.0218).

Whole-body TAG levels in wandering larvae reared from embryonic eclosion on the enriched diets revealed distinct effects of dietary composition on body fat content. When calculated per larva, there was no statistically significant change in TAG levels on the different diets, although the carbohydrate- and lipid-rich diets showed a trend towards increased TAGs (Fig 1A). However, when normalized to soluble protein content, TAG levels were significantly higher in animals fed a diet enriched in carbohydrates, and higher (but not quite statistically significantly so) on the lipid-rich diet (Fig 1B and 1C). I cannot exclude that more subtle, but biologically significant, changes in TAG content were missed due to a lack of sensitivity of our assay. Changes in soluble protein content per larva likely reflect changes in overall body mass (see below). To visualize differences in fat storage using an assay that does not require such normalization, I dissected the FBs from larvae and stained them with the lipophilic dye Nile Red [19]. As shown in Fig 1D, Nile-Red-staining material was found outside of the isolated FB tissues from larvae grown in 4C or 4L, whereas this was not observed in FBs from larvae fed the 4P or N diet. I interpret this apparent “leakage” of lipid from the 4C and 4L samples as indicative of elevated levels of lipids within the lipid droplets of these animals. Excess lipids inside lipid droplets and/or more or bigger lipid droplets inside FB cells may result in tissue fragility, leading to rupture and lipid release upon dissection and staining. Taken together, these data suggest that diets rich in carbohydrates and lipids promote accumulation of fat in *Drosophila* larvae.

**Fig 2:**
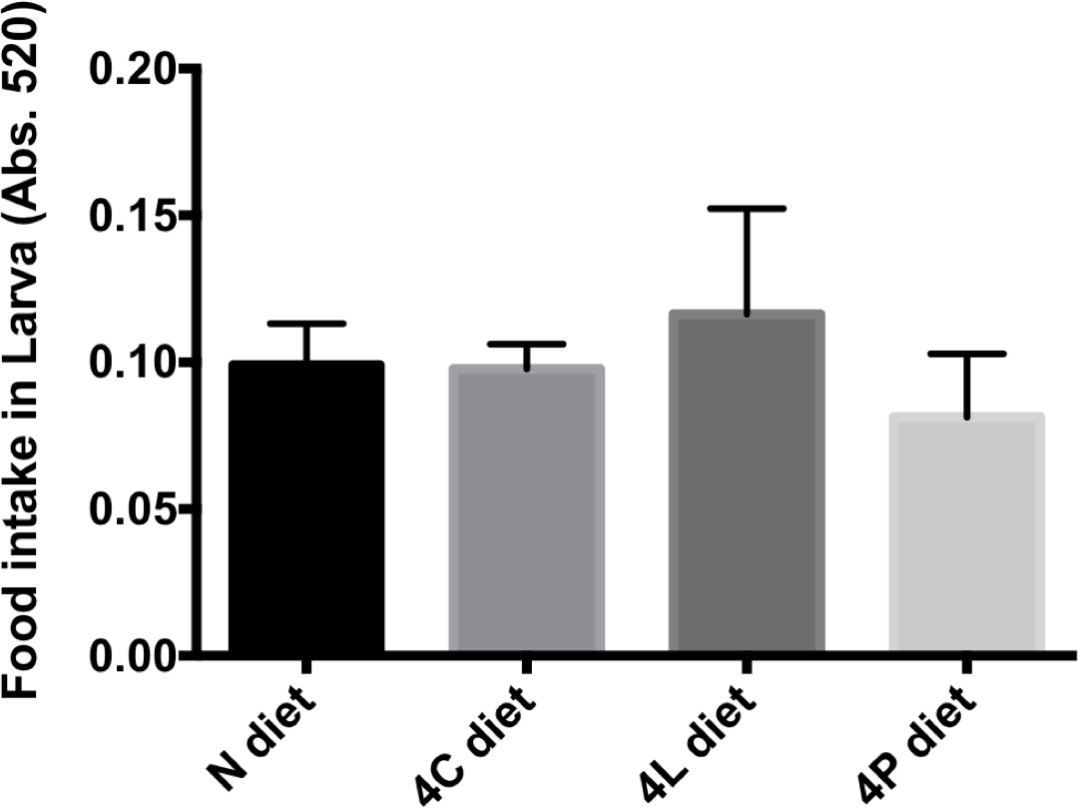
Food intake of flies fed enriched diets. Animals reared on the indicated diets were allowed to feed on red-colored food for a defined period of time. The amount of red food coloring in a homogenate was measured as absorbance at 520 nm. 20 larvae per diet were analyzed after a 30-min feeding period on the indicated synthetic diet. No significant differences where observed by Ordinary one-way ANOVA. Error bars, standard deviation.

Like other animals, fruit flies display food preferences based on taste [20], which could be altered significantly by dietary composition. Additionally, certain diets may trigger satiety signals more readily than do other diets. The increased fat observed in animals fed a carbohydrate-rich diet could thus reflect an increase in dietary intake or, alternatively, increased storage of dietary energy. I measured food consumption in larvae. Larvae that had hatched on the experimental diets were exposed for 30 min to the same diet to which colored dye was added [3]. By measuring the amount of food coloring in larval homogenate [3], I found a trend towards reduction in feeding for animals fed the P-rich diet, but this difference was not statistically significant (Fig 2). These findings are consistent with results from studies in other systems in which the protein content of diet appears to trigger satiety signals [10, 21, 22]. No differences were observed between the amounts of food ingested in a N, 4C or 4L diet (Fig 2). The lack of major differences in larval food consumption suggest that feeding behavior has at most a small contribution to the observed metabolic alterations in larvae fed the different diets (Fig 1).

**Fig 3:**
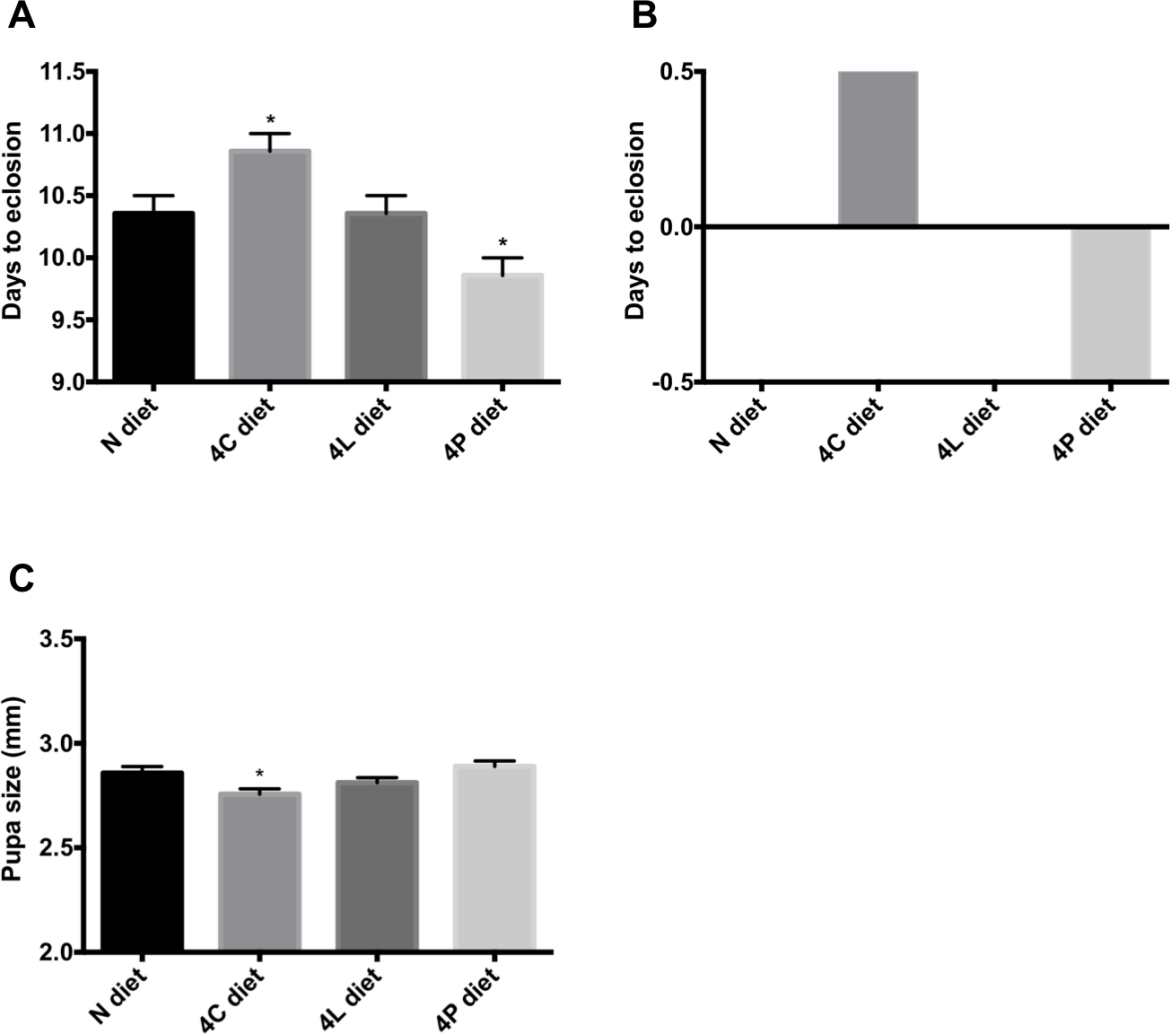
Effect of enriched diets on times to eclosion and larval size. (A) The duration (in days) from egg laying to eclosion of adult flies on the indicated diets. 7 independent experiments (totaling ≥ 230 animals per experimental condition) were performed. (B) The mean values in (A) were subtracted from the mean duration measured on the N diet. (C) Flies (*n* = 54 per condition) grown on the different diets were allowed to develop until pupa stages, at which time a caliper was used to measure their body length. Asterisks indicate significant differences (P < 0.05) for Ordinary one-way ANOVA. Error bars, standard error of the mean.

In many species, consumption of insufficient calories stunts development and results in smaller adults [23–26]. An imbalance of calorie sources also has adverse effects [25, 26]. I wondered if diets enriched in certain nutrients would alter rates of development and body size. Animals fed a 4P diet eclosed a half day faster than those fed the N diet (Fig 3A), whereas excess carbohydrates led to a half-day developmental delay (Fig 3A and 3B). 4L diet had no effect on developmental timing (Fig 3A and 3B). The majority of organismal growth occurs during larval development. To determine if prolonged or curtailed development on the 4P and 4C diets translated into differences in body size, I measured pupal length in animals raised on the different enriched diets (Fig 3C). Although 4P-fed flies eclosed faster than those on any another medium, their overall size was not affected (Fig 3C). On the other hand, slower-eclosing larvae fed a 4C diet made smaller pupae (Fig 3C), consistent with the decreased protein content of larvae on this diet (Fig 1B). The 4L diet had no effect on pupal size (Fig 3C). Thus, the alterations in the timing of development that I observed with the 4P and 4C diets are consistent with a model in which faster development indicates an abundance of available resources for growth, and slower development reflects a scarcity of such resources, with protein representing a better resource than carbohydrate.

**Fig 4:**
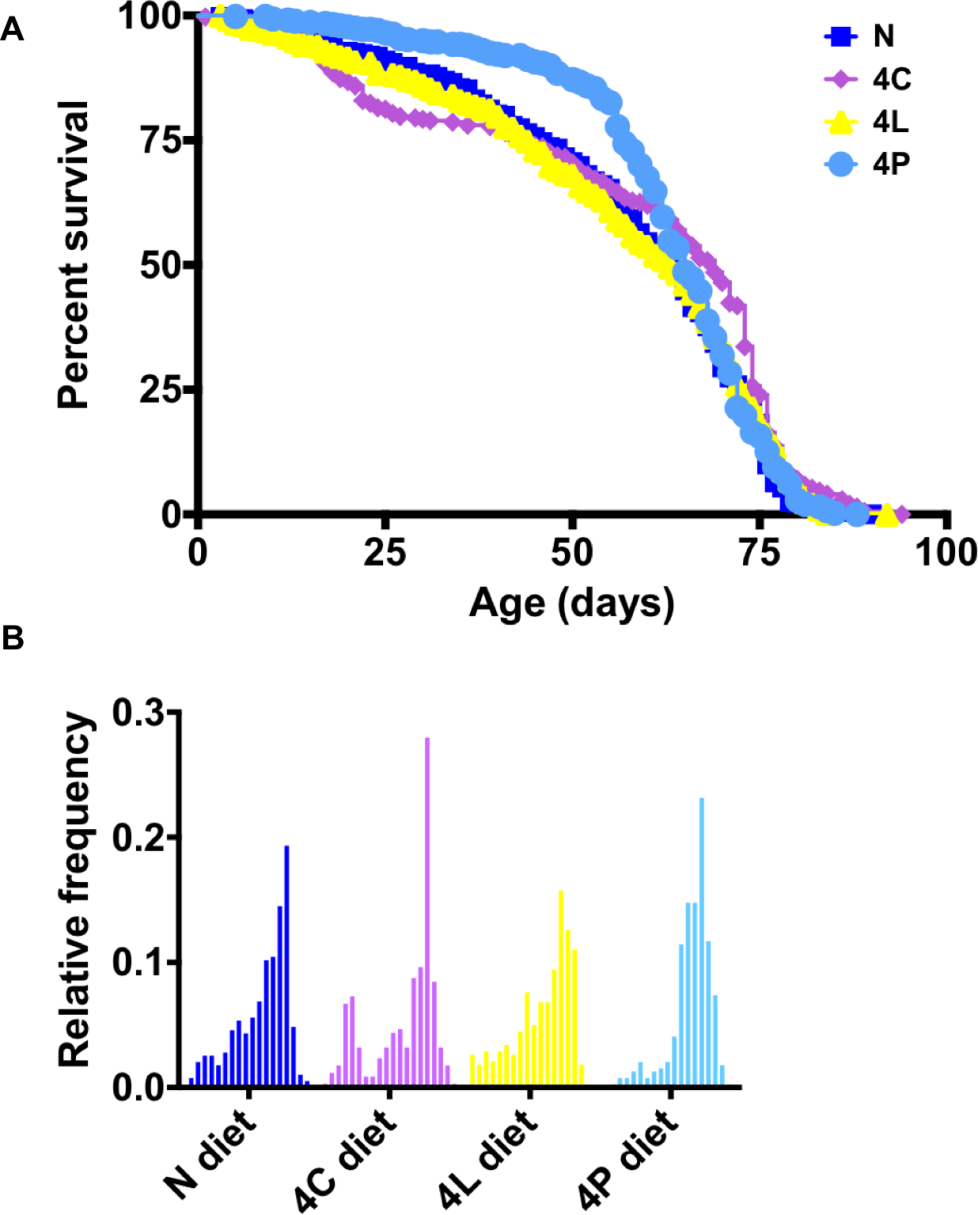
Effects of enriched diets on adult lifespan. Longevity of adult flies fed the indicated diets. *n*≥352 per diet. (A) Survival curves. Log-rank (Mantel-Cox) test indicates that the curves are significantly different (P = 0.0048). Median lifespans (days); N, 63; 4C, 69; 4L, 63; 4P, 65. (B) Histograms of individual lifespans.

It has been well documented that calorie intake influences lifespan [6]. In earlier studies using undefined media to which specific dietary components were added, varying the ratio of sugar to yeast had significant effects on lifespan [10, 23]. I also observed effects of synthetic media on adult lifespan (Fig 4A). Flies fed a 4C diet displayed an unusual bimodal distribution of lifespans composed of a short-lived population and a long-lived population (Fig 4B). No difference was observed between N and 4L diets. Comparing the N and 4P diet, however, I found that flies begin dying later on a 4P diet, with a median survival extended by two days compared to N diet (Fig 4), and a statistically significant difference in overall survival (Gehan-Breslow-Wilcoxon Test P = 0.0013). In *Drosophila* and most other species, reproduction limits lifespan, representing an apparent “trade-off” [27]. Notably, I monitored whether or not new adult animals eclosed in the vials in which the flies had laid their eggs over a period of 2–3 days, and found no apparent differences in fertility between any of these synthetic diets (data not shown).

## Discussion

This study differs in several important ways from others that have experimentally altered dietary content in *Drosophila*. Foremost among these is the progression of larval development on the N diet, which is only slightly delayed (0.5–1 day) relative to standard diets optimized to shorten the timing of development. Other defined diets strongly delay development (Table 1), and were optimized instead for reproducible lifespan analysis without compromising fecundity [12, 13]. On one such diet, developmental timing was delayed approximately two-fold, and only upon the addition of yeast extract–an undefined ingredient–could development be restored to within 1 day of that achieved on an undefined sugar-yeast diet [13]. Larval development is highly focused on the accumulation of energy to support metamorphosis and early adult life. *Drosophila* metabolism has presumably evolved under selective pressure to maintain efficient larval development, and it is thus important to consider this context in the study of gene-diet relationships. Importantly, egg laying was not significantly decreased on any enriched diet (S1 Fig). In fact, on the 4L diet adult females laid slightly more eggs (S1 Fig). However, the total number of eggs was low when compared to the number of eggs reported for other diets, suggesting that the synthetic diet is not likely to support high rates of egg laying.

Studies focused on *Drosophila* energy metabolism often utilize enriched diets, created by adding specific ingredients to an undefined diet. This approach has significant limitations. In mammalian systems, for example, the ability to test various ratios of carbohydrates to fat often requires reducing the levels of a single ingredient below the amount found in a “standard” diet, while maintaining standard levels of other ingredients [28]. Although I have not yet explored the effects of reducing dietary components, the diets described here facilitate this kind of approach in *Drosophila*.

Moreover, it is impossible to define the final concentration of a dietary component when a specific ingredient is simply added to an undefined diet. In a *Drosophila* model of diet-induced heart disease, dietary lipid content was increased by the addition of coconut oil, a lipid-rich (undefined) ingredient [9]. Minimal organismal effects were seen at concentrations below 30% coconut oil [9]. Considering that I was able to increase dietary lipid content four-fold without any noticeable adverse effects on lifespan or developmental timing, one can estimate that, in order to bring about the kinds of changes observed in “high-fat”/”high-lipid” diets based on 30% coconut oil, dietary lipid content must be increased by considerably more than four-fold, and/or the added source of lipids must contain higher proportions of specific storage-promoting molecules (e.g., TAGs). High-fat/high-lipid diets in human populations are unlikely to achieve a fat enrichment of this magnitude.

Studies with undefined diets found that increasing the proportional caloric contribution from yeast produced lean flies, whereas when the same number of calories were provided primarily from sugar, triacylglycerides were increased [10]. It was also found that the yeast:sucrose ratio was important for optimal life span, as increasing this ratio decreased life span [10, 23], but increased fecundity [10]. Something other than protein *per se* in dietary yeast must be the relevant component with regards to this ratio, as my 4P diet increased life span, despite a high protein:sucrose ratio. However, increased longevity on a high-protein, low-carbohydrate diet also does not appear to match recent findings with mice [29] and humans [30]. The abundance of individual amino acids has been shown by others to make important contributions to longevity [31], and thus is also an important variable to consider for future studies. For example, casein in the diets reported here could be replaced with specific combinations of individual amino acids.

The unusual bimodal distribution of lifespans in the 4C-fed adults could reflect two groups of individuals: one population succumbed to adverse effects of elevated fat experienced during development and early adult life, whereas others that survived long enough were able to benefit from certain old-age-specific advantages of extra stored energy. Indeed, recent studies in humans suggest that a dietary requirements change significantly with age [30].

In conclusion, the diets described here offer an additional option for the analysis of macronutrient effects on various parameters of organismal metabolism in *Drosophila*.

## Supporting Information

S1 Fig Effect of enriched diets on egg laying. Eggs laid per 20 females over a 4-hr interval. For each diet, data represent three independent egg collections each from two independent populations of adults (8–10 days old). Asterisks indicate significant differences (P < 0.05). Error bars, standard error of the mean. Since in the variances appeared unequal, both a nonparametric test (Mann Whitney) and a t test with Welch’s correction for unequal variances were applied to these data. The Mann Whitney test indicated a significant increase in egg laying on the 4L diet (P = 0.0247). All other P < 0.05.
(JPG)
