# Supplementary figures and images for: Effects of Synthetic Diets Enriched in Specific Nutrients on Drosophila Development, Body Fat, and Lifespan
Source: PLoS One. 2016 Jan 7;11(1):e0146758. doi: 10.1371/journal.pone.0146758 (PMC4704830; doi:10.1371/journal.pone.0146758)

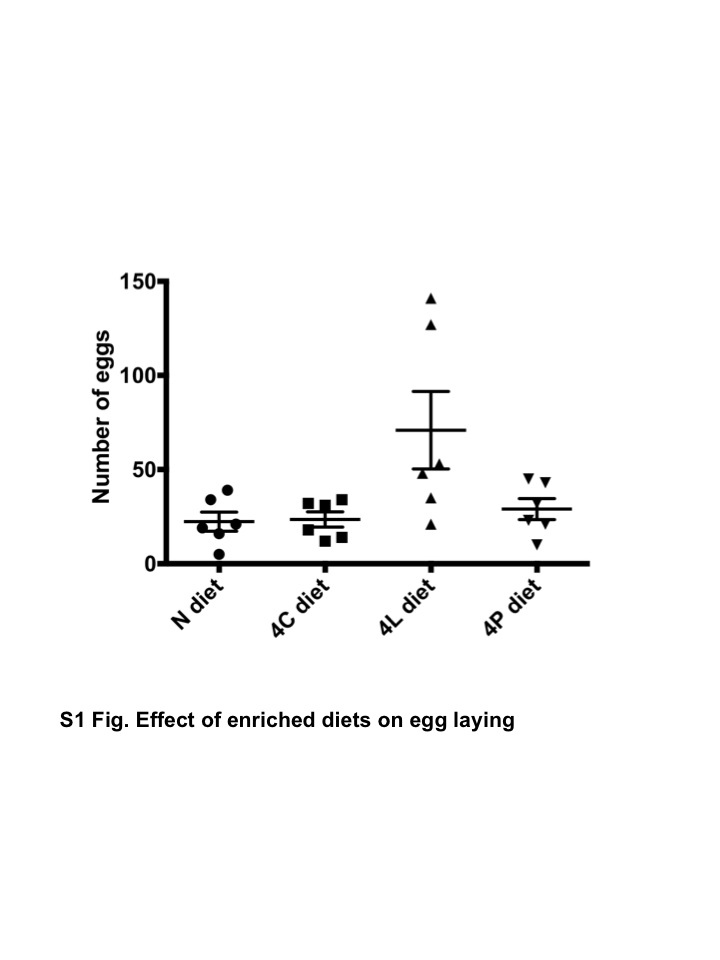

Supplement: S1 Fig — Eggs laid per 20 females over a 4-hr interval. For each diet, data represent three independent egg collections each from two independent populations of adults (8–10 days old). Asterisks indicate significant differences (P < 0.05). Error bars, standard error of the mean. Since in the variances appeared unequal, both a nonparametric test (Mann Whitney) and a t test with Welch’s correction for unequal variances were applied to these data. The Mann Whitney test indicated a significant increase in egg laying on the 4L diet (P = 0.0247). All other P < 0.05. (JPG) [file pone.0146758.s001.jpg]
